# Efficacy and Safety of CVT-E002, a Proprietary Extract of *Panax quinquefolius* in the Prevention of Respiratory Infections in Influenza-Vaccinated Community-Dwelling Adults: A Multicenter, Randomized, Double-Blind, and Placebo-Controlled Trial

**DOI:** 10.1155/2011/759051

**Published:** 2011-07-20

**Authors:** Janet E. McElhaney, Andrew E. Simor, Shelly McNeil, Gerald N. Predy

**Affiliations:** ^1^UBC Gerontology and Diabetes Research, 186-828 West 10th Avenue, Vancouver, BC, Canada V5Z 1L8; ^2^Department of Microbiology, Sunnybrook and Women's College Health Sciences Center, Toronto, QN, Canada M4N 3M5; ^3^Clinical Trials Research Center, IWK Health Center, 5850/5980 University Avenue, Halifax, NS, Canada B3K 6R8; ^4^Capital Health, Alberta Health Services, Suite 300, 10216-124 Street, Edmonton, AB, Canada T5N 4A3

## Abstract

CVT-E002 (a proprietary extract) was found to be effective in the prevention of upper respiratory infections (URIs) in healthy adults, and institutionalized and community-dwelling seniors. A multicenter, randomized, double-blind, placebo-controlled trial was carried out to determine effects of CVT-E002 in the prevention of URIs in influenza-vaccinated community-dwelling adults. 783 community-dwelling adults were randomized to receive placebo, 400 mg or 800 mg treatment/d (1 : 1 : 1) for 6 months. Primary analysis on the incidence of laboratory-confirmed-clinical URIs (LCCUs), including influenza A and B, was performed on those receiving at least one dose. Secondary analysis was performed on study completers and included incidence, severity, and duration of URIs meeting a Jackson-based criteria and safety of CVT-E002. The incidence of LCCUs in the ITT group was 5.5%, 5.2%, and 4.6% in the placebo, 400 mg and 800 mg groups, respectively (*P* = 0.89). Jackson-confirmed URIs were significantly lower in the treated groups (*P* < 0.04). CVT-E002 supplementation reduced the severity and duration of Jackson-confirmed URIs. The results indicate that CVT-E002 can be safely used by similar groups and may prevent symptoms of URIs; larger sample size is warranted.

## 1. Introduction

Viruses are important causes of morbidity and mortality in elderly patients, and respiratory viral infections, particularly influenza, remain a significant cause of death in community-dwelling older adults. Influenza vaccination may have limited efficacy in this population, possibly due to age-associated declines in both humoral and cell-mediated immune responses [1, 2]. Cases of influenza in influenza-vaccinated seniors represent vaccine failure, and a therapy that can enhance the anti-influenza response in influenza-vaccinated seniors would be of therapeutic benefit in this population. 

CVT-E002 (COLD-FX^®^), a patented poly-furanosyl-pyranosyl-saccharide-based extract of *Panax quinquefolius* is known to have immunomodulatory properties [3, 4]. Evidence from numerous *in vitro* and animal studies indicates that CVT-E002 acts through Toll-like receptors to stimulate both innate and acquired immunity and enhance immune responses to viral infections.For example, in a study of human peripheral blood mononuclear cells cultured with live influenza virus, CVT-E002 was found to enhance the production of IL-2 and IFN-*γ* [4]. In another study, CVT-E002 increased the expression of IFN-*γ* in natural killer (NK) cells cultured with influenza virus (Y. A. Jing, Internal report). Daily administration of CVT-E002 for 6 weeks to mice with viral-induced leukemia was found to increase the proportions of macrophage and NK cells in the bone marrow and spleen of treated animals [5]. Clinical efficacy of CVT-E002 has also been demonstrated for the prevention and treatment of viral upper respiratory infections (URIs), in particular, influenza, in healthy adults, and institutionalized and community-dwelling seniors [6–8]. 

The present study was the largest multicenter study to date to examine the efficacy of daily dosing of CVT-E002 in the prevention of URIs in community-dwelling seniors who had received current-season influenza vaccine. It was also the only study to date to systematically assess a full panel of viral causes for respiratory infection in seniors taking CVT-E002 preventatively and the first study to look at the impact of CVT-E002 dose on efficacy. It was hypothesized that regular supplementation of CVT-E002 during influenza season would maintain immune stimulation and, therefore, would reduce the incidence, severity, and duration of viral URIs in otherwise healthy influenza-vaccinated seniors. It was also hypothesized a dose response would be observed resulting in improved efficacy of 800 mg/d of CVT-E002 over that observed with 400 mg/d. The specific objectives of the study were (1) to determine the dose-related prophylactic efficacy of CVT-E002 in community-dwelling influenza-vaccinated seniors on the incidence of laboratory-confirmed-clinical URIs, (2) to evaluate the effects of CVT-E002 on the incidence and frequency of all URIs meeting a Jackson-based criteria, (3) to determine the efficacy of CVT-E002 supplementation on the severity and duration of URIs, and (4) to assess the safety and tolerability of chronic administration of two dosages of CVT-E002 in older adults.

## 2. Methods

### 2.1. Study Design

This study was a multicenter, randomized, double-blind, placebo-controlled, three-arm trial. Influenza-vaccinated, community-dwelling seniors ≥65 years were recruited through four different health regions in Canada located in Edmonton, AB, Toronto, ON, Vancouver, BC, and Halifax, NS. The study was approved by all 4 local site REBs and was registered in the online registry, http://Clinicaltrials.gov/ (identifier NCT00240461). The 6 month study periods extended from approximately the middle of October, 2005 through April 2006 and from the middle of October, 2006 through April 2007. Follow-ups continued until August of each year.

### 2.2. Participant Recruitment and Responsibilities

Recruitment of seniors was done by disseminating study details through information sessions and the distribution of study advertisements at local influenza immunization clinics and community seniors' centers and groups. Those interested were screened for inclusion/exclusion criteria by the study coordinator. Eligible participants included those seasonally vaccinated for influenza who were available for follow-up visits, and also willing and able to sign a written informed consent. Subjects with the following medical conditions were excluded: HIV infection, malignancy, unstable cardiovascular disease, renal abnormalities, pulmonary disease, acute or active chronic liver disease, neurologic or psychiatric disease, active tuberculosis, multiple sclerosis, bleeding disorders, or planned surgery over the course of the trial. Those currently taking immunosuppressive therapy, oral steroids at dose equivalent of prednisone 10 mg/day or more, phenelzine, pentobarbital, haloperidol, warfarin, and heparin were also excluded. Additionally, those with a history of alcohol/drug abuse or known allergies to ginseng were not allowed to participate. Use of natural health products while participating in the study was also not allowed.

Participants logged their daily dosing and recorded any concomitant medication(s) consumed during the study. In addition, they recorded any adverse events they experienced during the study. Because URIs were considered an experimental endpoint, they were not included in the adverse event listings. AEs classified as infections included bladder infections, bone infections, skin infections, and lower respiratory tract infections such as pneumonia. It should be noted that since pain and pulmonary AEs could refer to non-URI-related events (e.g., arthritis pain) and since the URI definition required multiple symptoms of a certain intensity and duration, there is not a direct correlation between AEs in these categories and the incidence of Jackson-confirmed URI. Subjects maintained the same dosing, even during an URI, and were told to try and avoid any additional medications for their URIs unless prescribed by their physician. Initially, a one-month supply of the medication was given to the subjects. Additional medications were distributed after 1 and 3 months participation. A final visit was scheduled at 6 months. During these scheduled visits, they returned their completed study logs and any leftover medication. In addition, blood pressure measurements were completed during these visits. 

They were also required to immediately call the study coordinator if they developed any symptoms of a respiratory infection. A nasopharyngeal swab was obtained by the study coordinator within 5 days of the onset of URI symptoms and tested for influenza A and B, parainfluenza 1, 2, and 3, respiratory syncytial virus, adenovirus, coronavirus, and rhinovirus. All specimens were processed by a central viral laboratory using standard virologic methods consisting of viral culture, PCR, and direct fluorescent antibody analysis of the swabs. Participants were contacted weekly during the study to ensure adherence to the study protocol.

### 2.3. Preparation and Assignment of CVT-E002/Placebo

The study agent was CVT-E002, a proprietary product of Afexa Life Sciences Inc., standardized using a patented dual fingerprinting technique—ChemBioPrint—to ensure chemical and pharmacological consistency [3]. The study compared the effects of two dosages of CVT-E002, 400, and 800 mg/day, with a placebo. The placebo capsules, containing microcrystalline cellulose (MCC), were indistinguishable in appearance, color, and size from the treatment capsules. The capsules contained either 100 mg CVT-E002 + 100 mg MCC or 200 mg CVT-E002 or 200 mg MCC. Capsules were packaged in identical bottles containing 125 capsules/bottle. The bottle labels were identical (patient ID number was the only differentiator between bottles). Subjects for whom consent had been obtained were randomized by the study coordinator(s) using a one-to-one-to-one allocation of placebo and the two CVT-E002 arms using block randomization (block size of 12) created by EGA Biosciences, an independent company. Treatment consisted of taking 2 capsules of study medication twice every day for 6 months following the onset of the influenza season. The timing of doses and relation between dosing and meals was not specified. Blinding could be broken in case of a serious adverse event by use of the treatment codes provided by EGA Biosciences to the investigators in individual sealed opaque envelopes.

### 2.4. URI Assessments

During an URI, subjects completed a daily log of the severity of their URI-related symptoms on a scale of 0–3 (0 = no symptom, 1 = mild symptom—no change in daily activities, 2 = moderate symptom—some decrease in ability to carry out daily activities, and 3 = severe symptom—confined to home or bed). The following symptoms were evaluated: sore throat, runny nose, nasal congestion, cough, myalgia, sneezing, ear aches, headache, fever, chills, and fatigue. Subjects completed their daily logs at approximately the same time each day. Daily symptom scores for all days in which a score exceeding 4 were reported were summed to calculate the total symptom score (TSS) [7, 9, 10]. 

The primary end point of the study was the incidence of acute respiratory illness referred to as laboratory-confirmed-clinical URIs over the 6-month study period. Laboratory confirmation was defined as a positive result for any of the viruses tested in the swab samples. Clinical infection was defined by the presence of at least two of the following symptoms: cough, headache, sore throat, and fever. Reported URIs were also evaluated using a validated symptom self-assessment tool. This assessment tool is referred to as Jackson criteria. Subjects were considered to have a Jackson-confirmed URI if the TSS over two successive days was greater than or equal to 14 and at least one of the following symptoms was present: sore throat, runny nose, nasal congestion, and cough [7, 11]. 

Adequacy of blinding was assessed by completion of a study medication perception questionnaire which subjects completed during their last visit to the center. Compliance was assessed by counting the capsules returned and was verified by checking the subjects' daily logs.

### 2.5. Statistical Analysis

Sample size was calculated assuming that the proportion of subjects with laboratory-confirmed-clinical URI in the control group would be 10% and that CVT-E002 supplementation at either dose would reduce the incidence to 3% or less. To detect an absolute reduction of 7% with 80% power at 5% significance level (two-sided), 194 subjects per group were required. To allow for multiple comparisons and loss to followup, a total of 260 subjects per group were enrolled, resulting in a total sample size of 780 subjects.

All analysis was performed by an independent statistician under blinded conditions. All randomized subjects who had received at least one dose of study medication were included in the primary efficacy analysis. This group was referred to as an intention-to-treat (ITT) population. Data were also analyzed for subjects completing the study. 

Between-group comparisons for the primary end point were made using the log-rank statistic for the time-to-infection approach for ITT group and using a chi-square test for comparing proportions for which only subjects completing the study were included.

The secondary efficacy variables included the 6-month rate of Jackson-confirmed URIs, severity and duration of URIs, and safety of chronic administration of CVT-E002. For the ITT group, between-group comparisons of the URIs were made using the log-rank statistic. For subjects completing the study, the rates of Jackson-confirmed URIs were compared using chi-square tests. The total symptom scores and the total days of infection in the three groups were compared using Wilcoxon two-sample tests. The incidence of adverse events, adverse events by category, and serious adverse events were compared using chi-square tests for comparing proportions. Between-group comparisons of measured compliance and the proportion of patients who were at least 80% compliant were made using an analysis of variance and a chi-square test, respectively.

The percentage of subjects in each treatment group that thought they were taking CVT-E002 and the percentage of subjects perceiving treatment to be effective were compared using chi-square tests for comparing proportions. SAS version 9.1 was used to perform the statistical analysis.

## 3. Results

### 3.1. Participants Characteristics

**Figure 1:**
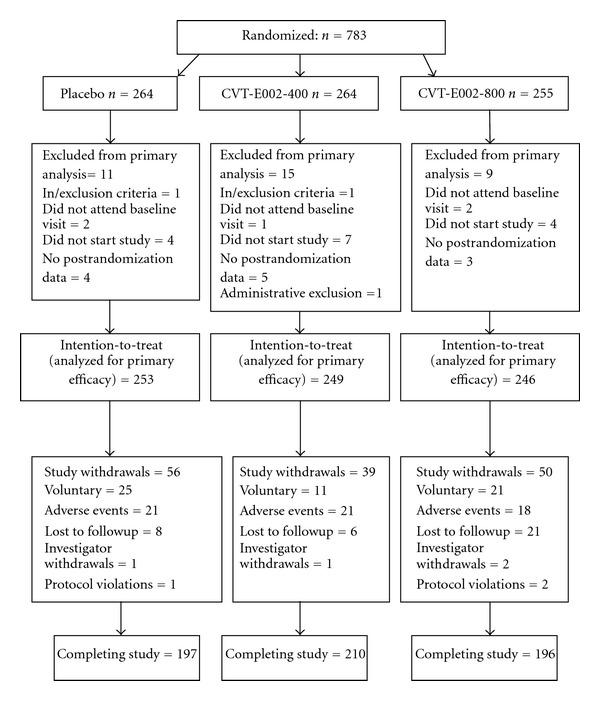
Disposition of the participants in the trial.

Following the screening process a total of 783 volunteers were recruited from 4 centers across Canada (Figure 1). Of these, 264, 264, and 255 received placebo, CVT-E002 400 mg/day, and CVT-E002 800 mg/day, respectively. Thirty five of these 783 subjects (11 from placebo group, 15 from CVT-E002-400, group and 9 from CVT-E002-800 group) were excluded from the analysis. Reasons for exclusion are described in Figure 1. A total of 748 subjects were included in the statistical analysis for primary efficacy endpoint, 253 in the placebo group, 249 in CVT-E002-400 group, and 246 in CVT-E002-800 group. A total of 145 subjects (19.4%), 56 (22.1%) in placebo group, 39 (15.7%) in CVT-E002-400 group, and 50 (20.3%) in CVT-E002-800 group withdrew from the study (Figure 1). Thus, a total of 197, 210, and 196 subjects in the placebo group, CVT-E002-400 group, and CVT-E002-800 group, respectively, completed the study (*P* = 0.17).

The three groups were found to be balanced in terms of age, gender, smoking status, weight, height, body mass index, and blood pressure (Table 1). Compliance was found to be similar among the groups in study completers (data not shown), however, significantly greater in the CVT-E002 groups than the placebo group in the ITT population (*P* = 0.036, Table 1).

### 3.2. Changes in URIs

The primary end-point in the study was the 6-month rate of lab-confirmed-clinical URIs. The 6-month rate of these URIs in the ITT group (*n* = 748) was found to be 5.5%, 5.2%, and 4.6% in the placebo, CVT-E002-400, and CVT-E002-800 group, respectively (*P* = 0.89, Table 2). For subjects completing the study (*n* = 603), the rate of laboratory-confirmed-clinical URIs was found to be 6.1%, 4.3%, and 4.6% in the placebo, CVT-E002-400, and CVT-E002-800 group, respectively (*P* = 0.67). The infection rates for the individual viruses tested in the study were found to be similar among the groups, with no statistically significant between-group differences seen (Table 3). 

 In addition to incidence of LCCU, incidence of clinically defined URI was examined. Use of clinical criteria for URI, such as a Jackson-based criteria, is valid and improvement in this type of measure is clinically relevant. The proportion of subjects in the ITT group (*n* = 748) with Jackson-confirmed URIs was 26.7% in the placebo group, 21.8% in the CVT-E002-400 group, and 19.3% in the CVT-E002-800 group (*P* < 0.23, Table 2). Because a previous study demonstrated that efficacy of CVT-E002 was only apparent in the final 8 weeks of a 16-week treatment period [6], an analysis was completed including only those participants who had completed the entire course of study. Among subjects completing the study (*n* = 603), the 180-day rate of Jackson-confirmed URIs was found to be 28.9% in the placebo group, 20.0% in the CVT-E002-400 group, and 19.4% in the CVT-E002-800 group (*P* < 0.04). Both the doses of CVT-E002 were found to significantly reduce the number of these URIs (*P* < 0.04 for 400 mg/day and *P* < 0.03 for 800 mg/day).

In subjects completing the study, the effects of the treatments on the number, duration, and severity of the URIs are also presented in Table 2. Overall between-group comparisons revealed that the treatments had no significant effects on the number, severity, or duration of lab-confirmed clinical URIs. However, the number of Jackson-confirmed URIs/subject was found to be significantly reduced in those taking CVT-E002 (*P* < 0.04). In addition, regular dosing of CVT-E002 at either 400 mg/day or 800 mg/day resulted in statistically significant reductions in symptom duration (total days of URI, *P* < 0.05) and symptom severity (TSS, *P* < 0.05) of the Jackson-confirmed URIs.

### 3.3. Adverse Events Reported

The incidence of all adverse events reported as well as serious adverse events is presented in Table 4. The percentage of subjects experiencing adverse events did not differ between the groups (*P* = 0.81). Statistical comparisons were also made by category. There were no among-group differences except for cardiovascular adverse events which were found to be significantly lower in the CVT-E002-400 and CVT-E002-800 groups (*P* = 0.05). A total of 36 subjects (4.8%) experienced serious adverse events during the study, 12 (4.7%) in the placebo group (myocardial infarction, hypotension, 2  × angina, 2  × gastroenteritis, 2  × bowel resection for polyp removal, appendectomy, hernia surgery, ataxia, prostate cancer, and severe pain in back and legs), 14 (5.6%) in the CVT-E002-400 group (atrial fibrillation, 2  × chest pain, fatal heart attack, 2  × gall bladder surgery, appendicitis, fractured arm, broken sternum, bone infection, prostate cancer, asthmatic bronchitis, cerebrovascular accident, and fainting), and 10 (4.1%) in the CVT-E002-800 group (pulmonary embolism, cardiac arrest, basal cell carcinoma, 2  × diarrhea and nausea, epistaxis, bruised ribs, chest pain, bladder infection, and mass in prostate) (*P* = 0.72). Of the SAEs, 6 were deemed possibly related to study medication: 2 in the placebo group (ataxia and gastroenteritis), 2 in the CVT-E002-400 group (asthmatic bronchitis and appendicitis), and 2 in the CVT-E002-800 group (pulmonary embolism and cardiac arrest). One fatal myocardial infarction, in the CVT-E002-400 group, was also reported and was deemed unlikely to be related to study medication. The treatment codes were broken for 2 subjects, one in the placebo group and one in the CVT-E002-400 group.

### 3.4. Perception of and Concomitant Medications

Subjects' perception of the study medication was also evaluated. Comparison of treatment groups revealed no differences in the percentage of those believing they were taking CVT-E002 (*P* = 0.45) or those perceiving the treatment to be effective (*P* = 0.58, data not shown). The proportion of subjects taking concomitant medications in different groups was found to be 98.0%, 97.2%, and 96.8% in the placebo, CVT-E002-400, and CVT-E002-800 groups, respectively (data not shown). 

## 4. Discussion

CVT-E002 (COLD-FX), a proprietary product of Afexa Life Sciences Inc., has been found to be effective in the prevention of URIs, including influenza, in a variety of populations including healthy adults, institutionalized seniors, and community-dwelling adults [6, 8]. The present study was designed to evaluate possible dose-related effects of CVT-E002 when administered to influenza-vaccinated, otherwise healthy, community-dwelling seniors (≥65 years). In particular, subjects with a seasonal immunization against influenza were recruited to determine if CVT-E002 supplementation would provide additional prophylaxis to that provided by the vaccine, which is not always protective in this population. The results indicate that regular supplementation of CVT-E002 during winter months was effective in reducing the number of Jackson-confirmed respiratory infections in healthy seniors. CVT-E002 treatment, however, was not found to have any significant effect on the incidence of laboratory-confirmed clinical URIs. These results are somewhat different from an earlier study in which regular dosing of CVT-E002 was found to be effective in reducing the incidence of acute respiratory illnesses due to influenza and RSV in influenza-vaccinated institutionalized seniors [8]. This discrepancy in the results could be explained, in part, by the lower than usual incidence rate of influenza reported at all 4 study sites during the present study. A report from Health Canada indicates that the overall percentage of influenza positive tests in the 2005-2006 season (8.5%) was lower than in the 2004-2005 and 2003-2004 seasons (12.7% and 12.3%, resp.) and a further lower incidence rate of 5.5% was observed in the present study (Table 2) [12, 13]. In addition, the age group accounting for the greatest proportion of cases during the 2005-2006 and 2006-2007 flu seasons was reported to be 0–14 age group (45%) as compared with the 2004-2005 season in which 46% of the cases were represented by the ≥65-year age group. In the present study, the milder nature of the influenza outbreaks and the lower rate of positivity for respiratory viral infections among participants (5.5%) may have resulted in an insufficient power to manifest treatment effects. Delays in collection and transportation of swab samples may also have contributed to the unexpectedly low rate of laboratory-confirmed infections and resulted in the lack of efficacy of CVT-E002 in meeting the primary end point. 

The design of this clinical trial was based on designs used to study products with direct impact on the virus. In the period of time since this clinical trial was designed, increasing knowledge about the mechanism of action of CVT-E002 has led to the understanding that CVT-E002 belongs to a new therapeutic class of polymolecular botanical drugs that impact the host immune system rather than the virus [14]. This new information indicates that endpoints, such as incidence of LCCU, designed to study efficacy of antiviral medications are not as relevant to the study of CVT-E002 as clinical endpoints are. 

Despite the insufficient number of laboratory-confirmed infections to validate primary outcome measures, CVT-E002 supplementation was found to affect secondary outcome measures significantly among subjects completing the study. The population of subjects completing the study was used in the analysis because of evidence from a previous trial that there is a threshold of time required for the treatment effect size to be measurable [6]. CVT-E002 was particularly effective in reducing the number of URIs meeting a Jackson-based criteria, a criteria based upon the subjects own perception of severity of the symptoms. Use of these clinical criteria of URI is valid and improvement in this measure is clinically relevant. CVT-E002 supplementation at 400 mg/day and 800 mg/day was found to reduce the relative risk of Jackson-confirmed URIs by 31% and 33%, respectively (Table 2). The number needed to treat with CVT-E002 doses of 400 mg/day and 800 mg/day was found to be 11.2 and 10.5, respectively. The TSS and duration of these URIs was also found to be reduced. The results are in agreement with an earlier study on healthy adults in which regular dosing of CVT-E002 during an influenza season was found to be effective in reducing the number, severity, and duration of Jackson-confirmed URIs [7]. Although the underlying mechanism(s) of these effects was not determined in the present study, evidence from earlier studies indicates that immune modulating properties of CVT-E002 may have been involved. CVT-E002 is known to act through Toll-like receptors to stimulate innate and Th1-type immune responses [3, 4, 14, 15]. It is, therefore, possible that by enhancing inflammatory responses of innate and T cells to the respiratory viruses, CVT-E002 may have reduced the viral load and hence prevented or shortened the duration of respiratory symptoms. The observed reduction in cardiovascular adverse events in participants taking CVT-E002 was unexpected and warrants further evaluation. 

The present study also compared the efficacy of two different doses of CVT-E002 on the incidence, duration, and severity of URIs in this high risk population. Despite the fact that the dose of 800 mg/day produced greater reduction in the number, severity, and duration of Jackson-confirmed URIs than the dose of 400 mg/day, the differences between these 2 groups were not significant. Both doses of CVT-E002 were safe and well tolerated when taken regularly as seasonal prophylaxis with no observed differences in the incidence of adverse events between subjects in the treatment and placebo arms and no treatment-related serious adverse events were observed.

In conclusion, although no significant between-group differences were seen for LCCUs, the data indicate that CVT-E002 at a dose of 400 mg/day or 800 mg/day is safe and well tolerated and results in a reduction in the number, severity, and duration of Jackson-confirmed URIs when taken as seasonal prophylaxis by healthy, community-dwelling older adults. Further studies with larger sample size are warranted to determine possible dose-related effects of CVT-E002.

## Figures and Tables

**Table 1:** Demographic and baseline characteristics and compliance by treatment group (intention-to-treat population).

Characteristic	Treatment group		
	Placebo (*n* = 253)	CVT-E002 400 mg (*n* = 249)	CVT-E002 800 mg (*n* = 246)
Age (years)	[232] 71.3 (5.5)	[244] 71.4 (5.5)	[233] 71.6 (7.5)
Sex—female	135/253 (53.4%)	143/249 (57.4%)	108/246 (43.9%)
Smoker	12/251 (4.8%)	15/246 (6.1%)	16/243 (6.6%)
Weight (kg)	[243] 75.9 (14.3)	[241] 75.0 (15.7)	[232] 77.5 (16.7)
Height (cm)	[243] 167.4 (10.3)	[241] 168.5 (11.2)	[233] 169.2 (9.9)
BMI	[243] 27.1 (4.7)	[241] 26.4 (5.1)	[232] 27.0 (5.3)

	Compliance		

mean (sd)	77.8 (34.2)	84.9 (27.8)	81.2 (30.5)^a^
Number >80% compliant	187 (73.9%)	202 (81.1%)	187 (76.0%)

For continuous variables entries represent mean (sd) and for dichotomous variables frequency/*n* (%). [*x*] denotes *n* for that measure if different than total.

^a^ *P* < 0.036 Between-group comparisons made using chi-square test for comparing proportions.

**Table 2:** Overall between-group comparison of infection rates, and severity and duration of URIs.

	Treatment groups			*P* value
	Placebo	CVT-E002-400 mg	CVT-E002-800 mg	
ITT (intention to treat)				
*n*	253	249	246	
Lab-confirmed clinical URIs	14 (5.5%)	13 (5.2%)	11 (4.5%)	0.89^a^
Jackson-confirmed URIs	67 (26.5%)	54 (21.7%)	47 (19.1%)	0.23^a^
Subjects completing the study				
*n*	197	210	196	
Lab-confirmed clinical URIs	12 (6.1%)	9 (4.3%)	9 (4.6%)	0.67^b^
Number of infections/subject	0.06 (0.24)	0.05 (0.26)	0.046 (0.21)	0.68^c^
Total days of infection	1.14 (5.3)	0.88 (4.6)	0.69 (4.2)	0.67^c^
Total symptom score	3.86 (17.7)	5.1 (29.1)	2.85 (15.1)	0.82^c^
Jackson-confirmed URIs	57 (28.9%)	42 (20.0%)	38 (19.4%)	0.04^b^
Number of URIs/subject	0.36 (0.64)	[209] 0.23 (0.50)	0.24 (0.56)	0.04^c^
Total days of URI	4.87 (11.2)	3.13 (7.9)	2.93 (7.2)	0.05^c^
Total symptom score	23.0 (49.6)	[209] 17.0 (43.3)	15.3 (38.0)	0.05^c^

^a^Between group comparisons made using log-rank test.

^b^Between-group comparisons made using chi-square test for comparing proportions.

^c^Between-group comparisons made using Wilcoxon two-sample test.

Jackson-confirmed URIs (subset analysis):

Total number: Placebo versus 400 mg, *P* < 0.04; versus 800 mg, *P* < 0.03; 400 mg versus 800 mg, *P* < 0.88.

Number of URIs/subject: placebo versus 400 mg, *P* < 0.04; versus 800 mg, *P* < 0.03; 400 mg versus 800 mg, *P* < 0.92.

Total days of URI: placebo versus 400 mg, *P* < 0.06; versus 800 mg, *P* < 0.05; 400 mg versus 800 mg, *P* < 0.89.

TSS: placebo versus 400 mg, *P* < 0.05; versus 800 mg, *P* < 0.04; 400 mg versus 800 mg, *P* < 0.92.

**Table 3:** Between group comparison in laboratory-confirmed infections for different viruses.

	Placebo	CVT-E002-400	CVT-E002-800	*P* value^a^
*n*	253	249	246	
Rhinovirus	4	9	8	0.338
Coronavirus OC 43	6	7	11	0.375
Coronavirus NL-63		1	3	0.164
Coronavirus 229-E		3	4	0.150
RSV	8	9	3	0.214
Parainfluenza	3	2	4	0.703
Influenza-A	2	3	1	0.609
Influenza-B			1	0.360
Adenovirus	1			0.376
Meta-pneumonia		4	2	0.131

^a^Between-group comparisons made using chi-square test for comparing proportions, all differences were found to be nonsignificant.

**Table 4:** Incidence of adverse events and serious adverse events among different groups.

	Treatment group			*P* value^a^
	Placebo (*n* = 253)	CVT-E002-400 (*n* = 249)	CVT-E002-800 (*n* = 246)	
*Adverse events*				
Total number of adverse events	215	220	216	
Any adverse event	117 (46.3%)	122 (49.0%)	119 (48.4%)	0.811
Adverse event by category				
Allergy	2 (0.8%)	0 (0.0%)	2 (0.8%)	0.478
Blood	1 (0.4%)	0 (0.0%)	0 (0.0%)	>0.999
Cardiovascular	17 (6.7%)	6 (2.4%)	9 (3.7%)	0.049
Constitutional	7 (2.8%)	8 (3.2%)	10 (4.1%)	0.715
Dermatology	12 (4.7%)	17 (6.8%)	10 (4.1%)	0.353
Endocrine	0 (0.0%)	2 (0.8%)	1 (0.4%)	0.328
Gastrointestinal	42 (16.6%)	30 (12.1%)	40 (16.3%)	0.284
Hemorrhage	1 (0.4%)	4 (1.6%)	3 (1.2%)	0.375
Infection	5 (2.0%)	5 (2.0%)	3 (1.2%)	0.828
Metabolic	0 (0.0%)	2 (0.8%)	0 (0.0%)	0.218
Musculoskeletal	8 (3.2%)	14 (5.6%)	6 (2.4%)	0.147
Neurology	10 (4.0%)	11 (4.4%)	15 (6.1%)	0.502
Ocular	1 (0.4%)	2 (0.8%)	2 (0.8%)	0.750
Oncology	1 (0.4%)	0 (0.0%)	2 (0.8%)	0.437
Otic	1 (0.4%)	1 (0.4%)	3 (1.2%)	0.461
Pain	28 (11.1%)	27 (10.8%)	24 (9.8%)	0.879
Pulmonary	32 (12.7%)	34 (13.7%)	34 (13.8%)	0.917
Renal/genitourinary	5 (2.0%)	8 (3.2%)	4 (1.6%)	0.460

*Serious adverse events*				
Total number of serious adverse events	13	14	10	
Any serious adverse event	12 (4.7%)	14 (5.6.0%)	10 (4.1%)	0.719
Serious adverse event by category				
Cardiovascular	4 (1.6%)	4 (1.6%)	2 (0.8%)	0.784
Dermatology	0 (0.0%)	0 (0.0%)	1 (0.4%)	0.329
Gastrointestinal	5 (2.0%)	3 (1.2%)	2 (0.8%)	0.623
Hemorrhage	0 (0.0%)	0 (0.0%)	1 (0.4%)	0.329
Infection	0 (0.0%)	1 (0.4%)	0 (0.0%)	0.662
Musculoskeletal	1 (0.4%)	2 (0.8%)	1 (0.4%)	0.849
Neurology	1 (0.4%)	2 (0.8%)	0 (0.0%)	0.550
Oncology	1 (0.4%)	0 (0.0%)	0 (0.0%)	>0.999
Pain	1 (0.4%)	0 (0.0%)	1 (0.4%)	0.775
Pulmonary	0 (0.0%)	1 (0.4%)	0 (0.0%)	0.662
Renal/genitourinary	0 (0.0%)	1 (0.4%)	2 (0.8%)	0.218

^a^When 50% of the cells have expected cell counts less than 5 Fisher's exact two tailed test was used. Otherwise, comparisons made using chi-square test for comparing proportions.

## Abbreviations List

ITT: Intention-to-treat
LCCU: Laboratory-confirmed clinical upper respiratory infection
MCC: Microcrystalline cellulose
REB: Research ethics board
Th1: T helper 1
TSS: Total symptom score
URI: Upper respiratory infection.
